# Trigger finger and the risk of systemic inflammatory and autoimmune rheumatic diseases: real-world evidence

**DOI:** 10.3389/fimmu.2026.1872187

**Published:** 2026-08-26

**Authors:** Zong-Han Lin, Yu-Jung Su, Chun-Ho Chen, Hui-Chin Chang, Shuo-Yan Gau

**Affiliations:** 1 Department of Orthopedics, Ditmanson Medical Foundation Chia-Yi Christian Hospital, Chia-Yi, Taiwan; 2 Orthopedics Department, Chi-Mei Medical Center, Tainan, Taiwan; 3 Department of Biomedical Engineering, National Cheng Kung University, Tainan, Taiwan; 4 Department of Orthopedics, School of Medicine, National Taiwan University, Taipei, Taiwan; 5 Evidence-based Medicine Center, Chung Shan Medical University Hospital, Taichung, Taiwan; 6 Library, Chung Shan Medical University Hospital, Taichung, Taiwan; 7 School of Medicine, Chung Shan Medical University, Taichung, Taiwan; 8 Institute of Allergology, Charité-Universitätsmedizin Berlin, Freie Universität Berlin and Humboldt-Universität zu Berlin, Berlin, Germany; 9 Department and Graduate Institute of Business Administration, National Taiwan University, Taipei, Taiwan; 10 Department of Medical Education, Ditmanson Medical Foundation Chia-Yi Christian Hospital, Chiayi, Taiwan

**Keywords:** cohort study, immunology, real world study, trigger finger, TriNetX

## Abstract

**Background:**

Trigger finger (TF) is a common hand disorder traditionally viewed as a localized mechanical condition. Recent evidences suggests potential links between tenosynovial pathology and systemic inflammatory diseases, especially rheumatoid arthritis, though large-scale longitudinal data remain limited.

**Methods:**

We conducted a retrospective cohort study using the TriNetX Global Collaborative Network. Adults diagnosed with TF between 2005 and 2018 were identified and compared with individuals undergoing routine health evaluations without TF. Patients with prior malignancy, systemic rheumatic diseases, or death before index were excluded. One-to-one propensity score matching balanced demographics, comorbidities, healthcare utilization, medications, and socioeconomic factors. The primary outcomes were incident systemic autoimmune and inflammatory rheumatic diseases, including inflammatory arthritis and connective tissue disorders. We estimated hazard ratios with 95% confidence intervals. Robustness was assessed through sensitivity analyses incorporating alternative matching strategies, extended washout periods, varying follow-up durations, and stricter exposure definitions, alongside subgroup analyses by age and sex. As validation, data from US Collaborative Network were also applied for analysis.

**Results:**

After matching, 82,566 patients were included in each cohort. TF was associated with increased risks of ankylosing spondylitis (HR 1.973, 95% CI 1.377–2.828), rheumatoid arthritis (HR 1.604, 95% CI 1.287–1.998), psoriatic arthritis (HR 1.832, 95% CI 1.428–2.352), and gout (HR 1.152, 95% CI 1.053–1.261). Elevated risks were also observed for systemic lupus erythematosus (HR 1.460, 95% CI 1.146–1.858) and Sjögren syndrome (HR 1.314, 95% CI 1.077–1.604). Associations remained consistent across most sensitivity analyses, including extended washout periods and alternative cohort definitions. Subgroup analyses demonstrated similar patterns across age and sex strata.

**Conclusions:**

TF was associated with systemic autoimmune and inflammatory rheumatic diseases. Current findings support a potential link between localized tendon pathology and broader systemic inflammatory processes.

## Introduction

Trigger finger (TF) is a prevalent hand pathology characterized by mechanical dysfunction during digital flexion and extension (1). The hallmark symptoms such as pain, snapping, and locking stem from a volumetric mismatch between the flexor tendons and the A1 pulley (2). This discrepancy impairs smooth tendon gliding and, in advanced stages, can culminate in fixed flexion deformities that severely diminish manual dexterity and patient quality of life (3). Originally interpreted from a purely mechanical perspective, the pathophysiology of TF is now understood as involving mechanical stress and degenerative changes, with metabolic dysfunction playing a causal role in disease development (4).

Current real-world data indicates that TF affects 2–3% of the general population, with a predilection for females in their fifth and sixth decades (1, 5). While the prevalence of TF rises to 10–20% in patients with diabetes mellitus, it is often managed as an isolated musculoskeletal event rather than being recognized as a potential manifestation of underlying metabolic disease (1, 6). Past studies have reported real-world associations between upper limb disorders and inflammatory diseases (7). Systemic autoimmune and inflammatory rheumatic diseases share certain inflammatory and immunologic features with localized tenosynovitis; however, existing evidence has largely focused on rheumatoid arthritis, and data regarding other rheumatic conditions remain limited. Given these overlapping pathways, a potential association between TF and subsequent development of systemic autoimmune and inflammatory rheumatic diseases is biologically plausible, but has not been systematically investigated.

To fill this gap in knowledge, we utilized the TriNetX Global Collaborative network to conduct a retrospective cohort study, and use another sub-database of TriNetX, the US collaborative network to provide additional validation. Our primary objective was to investigate the longitudinal risk of developing systemic autoimmune and inflammatory rheumatic diseases following a diagnosis of TF.

## Materials and methods

This investigation adopted a retrospective cohort framework using data sourced from the TriNetX Global Collaborative Network. The platform integrates continuously refreshed, de-identified electronic health records contributed by collaborative healthcare institutions worldwide and has been widely utilized in prior health economics and outcomes research (8–10). At present, it contains data from more than 130 participating organizations across the Americas, Europe, and Asia. To strengthen the reliability of the findings, an additional dataset from the US Collaborative Network was incorporated for additional validation. Ethical oversight for this research was secured from the Institutional Review Board of Chi Mei Medical Center, which approved the study protocol and waived the requirement for informed consent (Approval No.: 11502-E02). All procedures were carried out in alignment with the principles outlined in the Declaration of Helsinki.

Patient identification relied on administrative coding for diagnoses and prescribed medications, with detailed definitions provided in **Supplementary Table 1**. Patients were identified between January 1, 2005, and December 31, 2018. Individuals in the TF cohort were required to have at least two documented clinical visits with a diagnosis of TF, whereas the control cohort comprised individuals with at least two routine health examination records (ICD-10-CM Z00.00) and no history of TF. The index date was defined as the first qualifying encounter that fulfilled all eligibility criteria. Participants with any prior diagnosis of the study outcomes before the index date or any recorded history of malignancy or death in the database were excluded. To improve comparability between groups, 1:1 propensity score matching (PSM) was consistently implemented across all analyses.

Adjustment for potential confounding incorporated a broad range of factors spanning demographic characteristics, patterns of healthcare utilization and pre-existing comorbidities including hypertension, hyperlipidemia, diabetes mellitus, chronic ischemic heart disease, vitamin D deficiency, chronic kidney disease, liver disorders. Medication exposure (glucocorticoids and nonsteroidal anti-inflammatory agents), socioeconomic vulnerability, substance-related disorders, and family history of musculoskeletal or connective tissue diseases were also considered within the analytical model. The primary outcomes were newly identified systemic autoimmune and inflammatory rheumatic disorders during follow-up, categorized as follows:

- Inflammatory arthritis: ankylosing spondylitis, rheumatoid arthritis, psoriatic arthritis, gout
- Connective tissue diseases: systemic lupus erythematosus, Sjögren syndrome, dermatomyositis/polymyositis, systemic sclerosis

Rheumatoid arthritis was defined using ICD-10-CM code M05 (“*rheumatoid arthritis with rheumatoid factor”*), which was selected to improve diagnostic specificity. An additional sensitivity analysis evaluating seronegative rheumatoid arthritis (ICD-10-CM M06.0, “*rheumatoid arthritis with rheumatoid factor*”) was subsequently performed. Robustness of the findings was examined through multiple sensitivity analyses that applied alternative matching strategies, varied definitions of TF, and the introduction of washout and follow-up intervals designed to reduce the likelihood of reverse causation (detailed information reported in **Supplementary Table 2**). Further validation was achieved through the use of negative control outcomes. Additional subgroup analyses explored potential differences by age and sex.

All statistical procedures were conducted using the analytical tools embedded within the TriNetX platform. Risk comparisons between the TF and control cohorts were expressed as hazard ratios (HR) accompanied by 95% confidence intervals (95% CI). Baseline balance between groups was assessed using standardized mean differences (SMD), with values below 0.1 interpreted as indicating negligible imbalance. Follow-up commenced after a 3-month washout period following the index date and continued for 5 years in the primary and subgroup analyses. Sensitivity analyses applied alternative follow-up durations only when explicitly specified.

## Results

### Baseline characteristics

**Figure 1:**
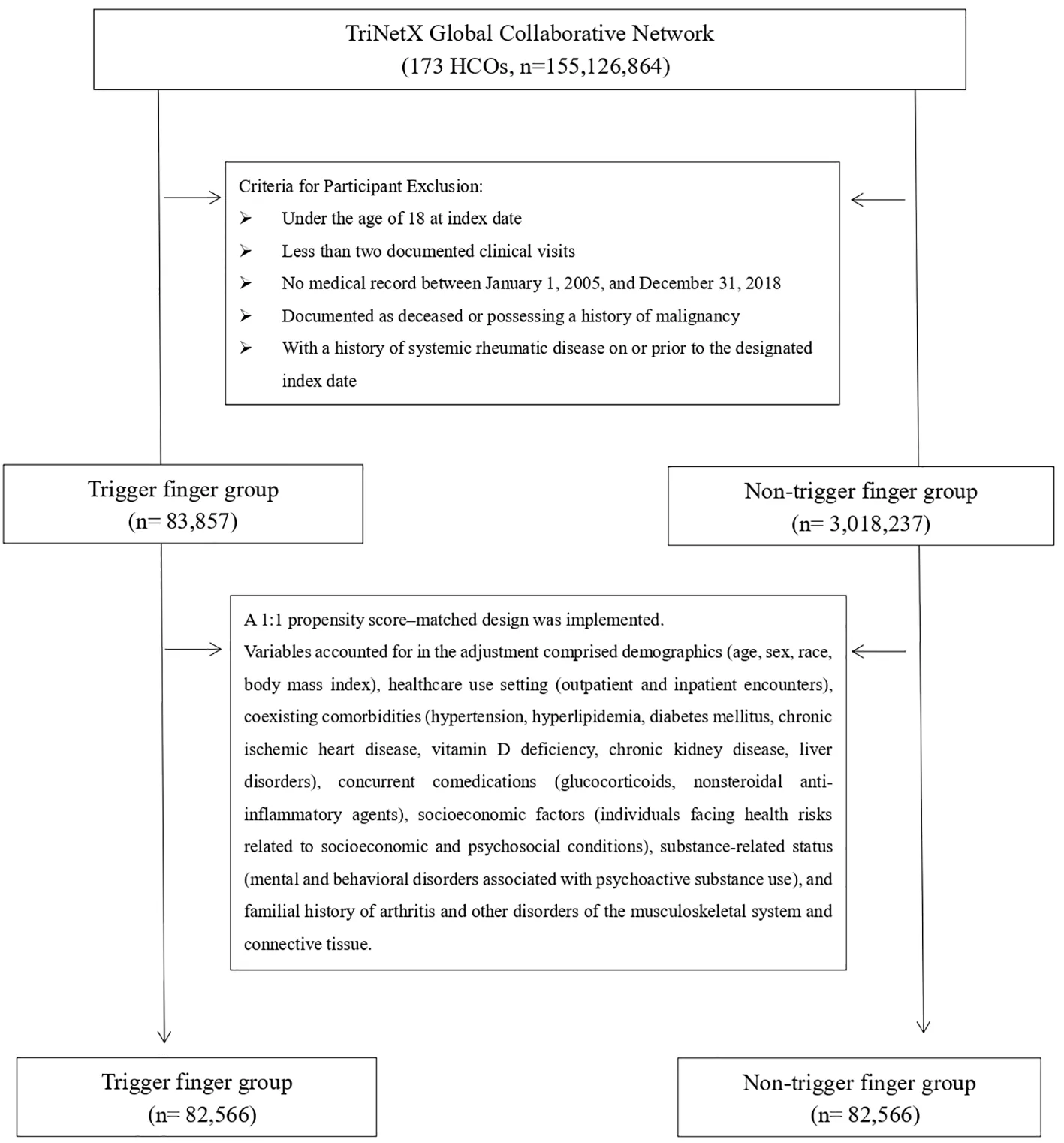
Patient selection flowchart.

A total of 83,857 patients with TF and 3,018,237 TF-free controls were initially identified (**Figure 1**). Before PSM, patients with TF were older (mean age 55.9 ± 15.2 vs 40.5 ± 17.9 years; SMD = 0.92) and more likely to be female (63.8% vs 56.6%; SMD = 0.15). In addition, the TF cohort had a higher prevalence of metabolic and cardiovascular comorbidities, including hypertension (27.4% vs 13.9%; SMD = 0.34), diabetes mellitus (15.7% vs 5.3%; SMD = 0.35), and hyperlipidemia (18.1% vs 8.7%; SMD = 0.28). After 1:1 PSM, 82,566 patients were included in each cohort, and baseline characteristics were well balanced, with all standardized mean differences below 0.1. The mean age was comparable between the two groups (55.9 ± 15.2 vs 56.2 ± 15.6 years; SMD = 0.02), and the sex distribution was similar (female: 63.8% vs 63.4%; SMD = 0.01). Distributions of race, comorbidities, socioeconomic status, healthcare utilization, and concomitant medications were also well matched (**Table 1**).

**Table 1:** Baseline characteristics.

	Before matching			After matching		
	TF cohort (n= 83,857)	Control cohort (n= 3,018,237)	SMD	TF cohort (n= 82,566)	Control cohort (n= 82,566)	SMD
Age at index						
Mean ± SD	55.9 ± 15.2	40.5 ± 17.9	0.92	55.9 ± 15.2	56.2 ± 15.6	0.02
Sex						
Female	52687 (63.8)	1695875 (56.6)	0.15	52687 (63.8)	52311 (63.4)	0.01
Male	29822 (36.1)	1281256 (42.8)	0.14	29822 (36.1)	30189 (36.6)	0.01
Unknown Gender	57 (0.1)	16860 (0.6)	0.09	57 (0.1)	66 (0.1)	0.00
Race, n (%)						
White	47935 (58.1)	1935616 (64.7)	0.14	47935 (58.1)	48751 (59.0)	0.02
Black or African American	10974 (13.3)	451320 (15.1)	0.05	10974 (13.3)	10990 (13.3)	0.00
Asian	6338 (7.7)	147975 (4.9)	0.11	6338 (7.7)	6121 (7.4)	0.01
American Indian or Alaska Native	314 (0.4)	10523 (0.4)	0.00	314 (0.4)	327 (0.4)	0.00
Native Hawaiian or Other Pacific Islander	499 (0.6)	12004 (0.4)	0.03	499 (0.6)	516 (0.6)	0.00
Other Race	2905 (3.5)	124129 (4.1)	0.03	2905 (3.5)	4919 (6.0)	0.12
Unknown Race	13601 (16.5)	312424 (10.4)	0.18	13601 (16.5)	10942 (13.3)	0.09
BMI, n (%)						
≧ 25 (kg/m^2^)	28809 (34.9)	796532 (26.6)	0.18	28809 (34.9)	28996 (35.1)	0.00
Medical Utilization Status, n (%)						
Visit: Ambulatory	67172 (81.4)	2312110 (77.2)	0.10	67172 (81.4)	67757 (82.1)	0.02
Visit: Inpatient Encounter	15974 (19.3)	314638 (10.5)	0.25	15974 (19.3)	15311 (18.5)	0.02
Socioeconomic status, n (%)						
Persons with potential health hazards related to socioeconomic and psychosocial circumstances	815 (1.0)	36116 (1.2)	0.02	815 (1.0)	693 (0.8)	0.02
Lifestyle, n (%)						
Mental and behavioral disorders due to psychoactive substance use	6290 (7.6)	173975 (5.8)	0.07	6290 (7.6)	6239 (7.6)	0.00
Family history, n (%)						
Family history of arthritis and other diseases of the musculoskeletal system and connective tissue	198 (0.2)	3243 (0.1)	0.03	198 (0.2)	160 (0.2)	0.01
Comorbidities, n (%)						
Hypertension	22601 (27.4)	417319 (13.9)	0.34	22601 (27.4)	22901 (27.7)	0.01
Hyperlipidemia	14964 (18.1)	259874 (8.7)	0.28	14964 (18.1)	14748 (17.9)	0.01
Diabetes mellitus	12965 (15.7)	157458 (5.3)	0.35	12965 (15.7)	12488 (15.1)	0.02
Vitamin D deficiency	5334 (6.5)	110511 (3.7)	0.13	5334 (6.5)	5053 (6.1)	0.01
Chronic ischemic heart disease	4459 (5.4)	60031 (2.0)	0.18	4459 (5.4)	4274 (5.2)	0.01
Diseases of liver	2236 (2.7)	36537 (1.2)	0.11	2236 (2.7)	2063 (2.5)	0.01
Chronic kidney disease	1895 (2.3)	29628 (1.0)	0.10	1895 (2.3)	1774 (2.1)	0.01
Comedications, n (%)						
Non-steroidal anti-inflammatory analgesics	10330 (12.5)	301648 (10.1)	0.08	10330 (12.5)	9569 (11.6)	0.03
Glucocorticoids	21744 (26.3)	437967 (14.6)	0.29	21744 (26.3)	21451 (26.0)	0.01

TF, TF; A 1:1 propensity score–matched design was implemented in each analysis. Variables accounted for in the adjustment comprised demographics (age, sex, race, body mass index), healthcare use setting (outpatient and inpatient encounters), coexisting comorbidities (hypertension, hyperlipidemia, diabetes mellitus, chronic ischemic heart disease, vitamin D deficiency, chronic kidney disease, liver disorders), concurrent comedications (glucocorticoids, nonsteroidal anti-inflammatory agents), socioeconomic factors (individuals facing health risks related to socioeconomic and psychosocial conditions), substance-related status (mental and behavioral disorders associated with psychoactive substance use), and familial history of arthritis and other disorders of the musculoskeletal system and connective tissue.

### Primary outcomes: risk of systemic inflammatory and autoimmune rheumatic diseases

**Figure 2:**
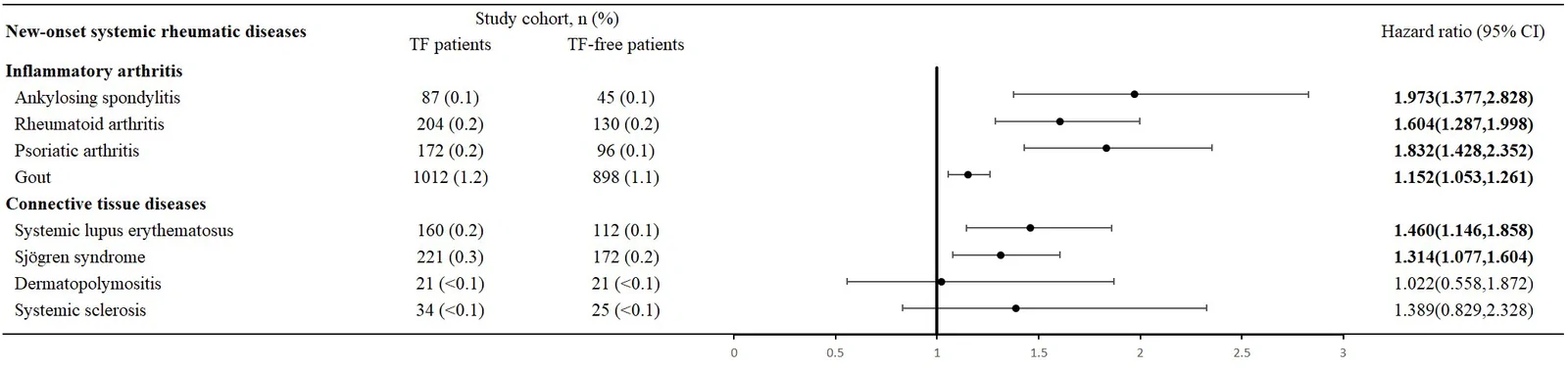
Risk of systemic inflammatory and autoimmune rheumatic diseases in TF patients under the structure of global collaborative network.

**Figure 3:**
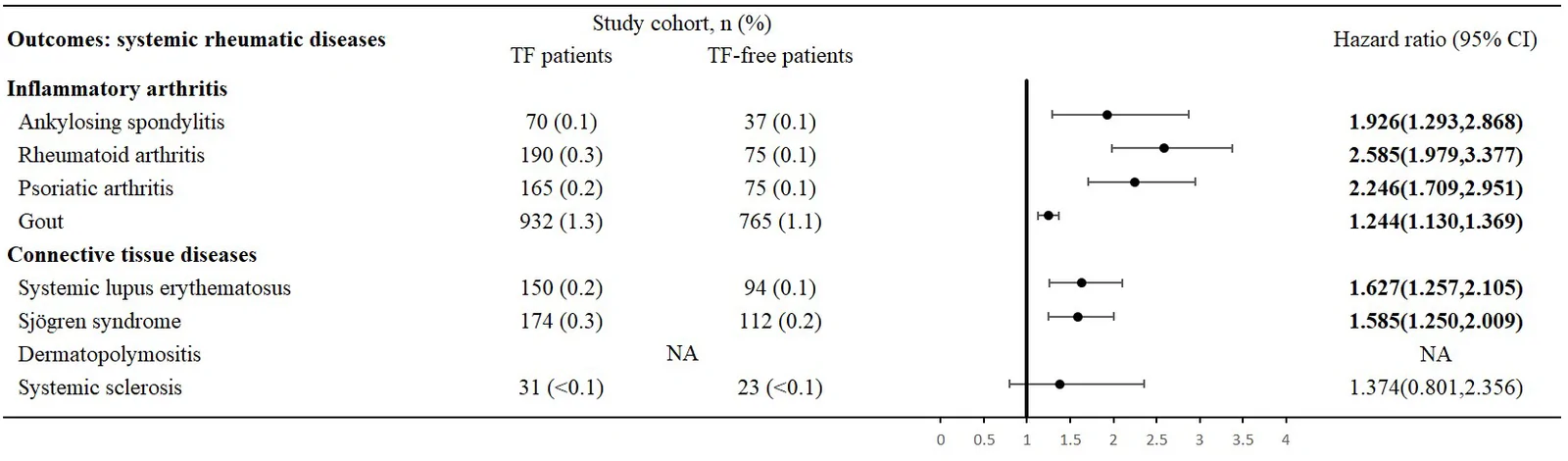
Risk of systemic inflammatory and autoimmune rheumatic diseases in TF patients under the structure of US collaborative network.

In the Global Collaborative Network, patients with TF exhibited a significantly increased risk of several systemic inflammatory and autoimmune rheumatic diseases compared with matched controls (**Figure 2**). Among inflammatory arthritis outcomes, TF was associated with a higher risk of ankylosing spondylitis (HR: 1.973; 95% CI: 1.377–2.828), rheumatoid arthritis (HR: 1.604; 95% CI: 1.287–1.998), and psoriatic arthritis (HR: 1.832; 95% CI: 1.428–2.352). A modest but statistically significant increase was also observed for gout (HR: 1.152; 95% CI: 1.053–1.261). For connective tissue diseases, TF was associated with an increased risk of systemic lupus erythematosus (HR: 1.460; 95% CI: 1.146–1.858) and Sjögren syndrome (HR: 1.314; 95% CI: 1.077–1.604). No statistically significant associations were observed for dermatopolymyositis (HR: 1.022; 95% CI: 0.558–1.872) or systemic sclerosis (HR: 1.389; 95% CI: 0.829–2.328). Consistent findings were also observed in the US Collaborative Network (**Figure 3**). To assess potential bias, negative outcome analyses were performed (**Supplementary Table 3**). No significant associations were observed between TF and infectious mononucleosis (HR: 0.708; 95% CI: 0.433–1.156) or acute appendicitis (HR: 0.982; 95% CI: 0.786–1.227), supporting the specificity and robustness of the primary findings.

### Sensitivity analyses

When restricting the follow-up period to 10 and 15 years, the associations between TF and systemic rheumatic diseases remained consistent (**Table 2**). Specifically, TF was associated with increased risks of ankylosing spondylitis (10-year: HR 1.805, 95% CI 1.384–2.354; 15-year: HR 1.746, 95% CI 1.355–2.249), rheumatoid arthritis (HR 1.614, 95% CI 1.368–1.905; HR 1.709, 95% CI 1.459–2.002), and psoriatic arthritis (HR 1.659, 95% CI 1.371–2.009; HR 1.638, 95% CI 1.363–1.967). Elevated risks were also observed for systemic lupus erythematosus (HR 1.388, 95% CI 1.145–1.682; HR 1.421, 95% CI 1.181–1.710) and Sjögren syndrome (HR 1.255, 95% CI 1.079–1.459; HR 1.292, 95% CI 1.118–1.493), while gout showed a modest but consistent increase (HR 1.119, 95% CI 1.043–1.200; HR 1.133, 95% CI 1.059–1.212). Analyses applying extended wash-out periods of 12 and 24 months also observed similar results (**Table 3**). TF remained associated with higher risks of ankylosing spondylitis (HR 2.135, 95% CI 1.443–3.160; HR 2.041, 95% CI 1.335–3.120), rheumatoid arthritis (HR 1.518, 95% CI 1.215–1.897; HR 1.457, 95% CI 1.156–1.836), and psoriatic arthritis (HR 1.791, 95% CI 1.388–2.311; HR 2.115, 95% CI 1.596–2.802). Associations with systemic lupus erythematosus also persisted (HR 1.446, 95% CI 1.123–1.861; HR 1.811, 95% CI 1.364–2.403), whereas findings for Sjögren syndrome were attenuated and became non-significant in the 24-month wash-out model (HR 1.216, 95% CI 0.968–1.527).

**Table 2:** Sensitivity analysis assessing the risk of incident systemic inflammatory and autoimmune rheumatic diseases in patients with TF under varying post-index follow-up durations.

Outcomes	Up to 10 years			Up to 15 years		
	TF cohort (%)	Control cohort (%)	HR (95% CI)	TF cohort (%)	Control cohort (%)	HR (95% CI)
Inflammatory arthritis						
Ankylosing spondylitis	152 (0.2)	85 (0.1)	**1.805 (1.384,2.354)**	165 (0.2)	94 (0.1)	**1.746 (1.355,2.249)**
Rheumatoid arthritis	365 (0.4)	228 (0.3)	**1.614 (1.368,1.905)**	417 (0.5)	242 (0.3)	**1.709 (1.459,2.002)**
Psoriatic arthritis	277 (0.3)	169 (0.2)	**1.659 (1.371,2.009)**	302 (0.4)	184 (0.2)	**1.638 (1.363,1.967)**
Gout	1632 (2.0)	1476 (1.8)	**1.119 (1.043,1.200)**	1822 (2.2)	1603 (1.9)	**1.133 (1.059,1.212)**
Connective tissue diseases						
Systemic lupus erythematosus	247 (0.3)	180 (0.2)	**1.388 (1.145,1.682)**	272 (0.3)	191 (0.2)	**1.421 (1.181,1.710)**
Sjögren syndrome	380 (0.5)	306 (0.4)	**1.255 (1.079,1.459)**	423 (0.5)	326 (0.4)	**1.292 (1.118,1.493)**
Dermatopolymositis	36 (<0.1)	30 (<0.1)	1.217 (0.750,1.976)	42 (<0.1)	31 (<0.1)	1.352 (0.850,2.151)
Systemic sclerosis	49 (0.1)	44 (0.1)	1.128 (0.751,1.694)	59 (0.1)	50 (0.1)	1.164 (0.798,1.697)

TF, TF; 95% CI, 95% confidence interval; HR, hazard ratio. In the TriNetX analytics platform, deidentification rules require that any incident case count of 10 or fewer be reported as 10.

A 1:1 propensity score–matched design was implemented in each analysis. Variables accounted for in the adjustment comprised demographics (age, sex, race, body mass index), healthcare use setting (outpatient and inpatient encounters), coexisting comorbidities (hypertension, hyperlipidemia, diabetes mellitus, chronic ischemic heart disease, vitamin D deficiency, chronic kidney disease, liver disorders), concurrent comedications (glucocorticoids, nonsteroidal anti-inflammatory agents), socioeconomic factors (individuals facing health risks related to socioeconomic and psychosocial conditions), substance-related status (mental and behavioral disorders associated with psychoactive substance use), and familial history of arthritis and other disorders of the musculoskeletal system and connective tissue. The bold values indicates statistically significance.

**Table 3:** Sensitivity analysis assessing the risk of incident systemic inflammatory and autoimmune rheumatic diseases in patients with TF under varying wash-out periods.

Outcomes	12 months			24 months		
	TF cohort (%)	Control cohort (%)	HR (95% CI)	TF cohort (%)	Control cohort (%)	HR (95% CI)
Inflammatory arthritis						
Ankylosing spondylitis	77 (0.1)	37 (<0.1)	**2.135 (1.443,3.160)**	64 (0.1)	32 (<0.1)	**2.041 (1.335,3.120)**
Rheumatoid arthritis	192 (0.2)	130 (0.2)	**1.518 (1.215,1.897)**	174 (0.2)	122 (0.1)	**1.457 (1.156,1.836)**
Psoriatic arthritis	162 (0.2)	93 (0.1)	**1.791 (1.388,2.311)**	149 (0.2)	72 (0.1)	**2.115 (1.596,2.802)**
Gout	961 (1.1)	811 (1.0)	**1.218 (1.109,1.338)**	835 (0.9)	731 (0.8)	**1.167 (1.057,1.289)**
Connective tissue diseases						
Systemic lupus erythematosus	145 (0.2)	103 (0.1)	**1.446 (1.123,1.861)**	133 (0.1)	75 (0.1)	**1.811 (1.364,2.403)**
Sjögren syndrome	199 (0.2)	166 (0.2)	**1.232 (1.002,1.513)**	162 (0.2)	136 (0.2)	1.216 (0.968,1.527)
Dermatopolymositis	18 (<0.1)	20 (<0.1)	0.925 (0.489,1.749)	12 (<0.1)	11 (<0.1)	1.113 (0.491,2.522)
Systemic sclerosis	31 (<0.1)	27 (<0.1)	1.179 (0.704,1.975)	27 (<0.1)	25 (<0.1)	1.103 (0.640,1.900)

TF, TF; 95% CI, 95% confidence interval; HR, hazard ratio. In the TriNetX analytics platform, deidentification rules require that any incident case count of 10 or fewer be reported as 10.

A 1:1 propensity score–matched design was implemented in each analysis. Variables accounted for in the adjustment comprised demographics (age, sex, race, body mass index), healthcare use setting (outpatient and inpatient encounters), coexisting comorbidities (hypertension, hyperlipidemia, diabetes mellitus, chronic ischemic heart disease, vitamin D deficiency, chronic kidney disease, liver disorders), concurrent comedications (glucocorticoids, nonsteroidal anti-inflammatory agents), socioeconomic factors (individuals facing health risks related to socioeconomic and psychosocial conditions), substance-related status (mental and behavioral disorders associated with psychoactive substance use), and familial history of arthritis and other disorders of the musculoskeletal system and connective tissue. The bold values indicates statistically significance.

Sensitivity analyses using different matching strategies further supported the main findings (**Table 4**). In the crude model without propensity score matching, associations were observed across most outcomes, including rheumatoid arthritis (HR 3.247, 95% CI 2.815–3.745) and psoriatic arthritis (HR 2.839, 95% CI 2.432–3.314). After matching on age and sex (Model 1), the associations remained significant, although with attenuated effect sizes, such as rheumatoid arthritis (HR 1.884, 95% CI 1.498–2.370) and systemic lupus erythematosus (HR 1.639, 95% CI 1.278–2.102). Similar results were observed when trigger finger was more stringently defined by the coexistence of a trigger finger diagnosis and a tendon sheath injection procedure (CPT code 20550). The associations with ankylosing spondylitis, rheumatoid arthritis, psoriatic arthritis, gout, and Sjögren syndrome remained statistically significant. Analyses for dermatomyositis/polymyositis and systemic sclerosis could not be performed because of insufficient outcome events, whereas the association with systemic lupus erythematosus was no longer statistically significant because of the limited number of events (**Table 5**). TF was associated with increased risks of ankylosing spondylitis (HR 1.897, 95% CI 1.235–2.914), rheumatoid arthritis (HR 2.183, 95% CI 1.685–2.828), psoriatic arthritis (HR 2.933, 95% CI 2.170–3.963), gout (HR 1.377, 95% CI 1.243-1.525) systemic lupus erythematosus (HR 1.994, 95% CI 1.480–2.688), and Sjögren syndrome (HR 1.622, 95% CI 1.297–2.028). Similar results were observed when seronegative rheumatoid arthritis (ICD-10-CM M06.0) was evaluated separately, with TF remaining significantly associated with an increased risk (HR, 2.945; 95% CI, 2.244–3.864) (**Supplementary Table 4**).

**Table 4:** Sensitivity analysis assessing the risk of incident systemic inflammatory and autoimmune rheumatic diseases in patients with TF under varying matching strategies.

Outcomes	Crude model			Model 1		
	TF cohort (%)	Control cohort (%)	HR (95% CI)	TF cohort (%)	Control cohort (%)	HR (95% CI)
Inflammatory arthritis						
Ankylosing spondylitis	88 (0.1)	1179 (<0.1)	**2.527 (2.035,3.138)**	88 (0.1)	46 (0.1)	**1.965 (1.375,2.806)**
Rheumatoid arthritis	207 (0.2)	2157 (0.1)	**3.247 (2.815,3.745)**	207 (0.2)	113 (0.1)	**1.884 (1.498,2.370)**
Psoriatic arthritis	174 (0.2)	2076 (0.1)	**2.839 (2.432,3.314)**	174 (0.2)	96 (0.1)	**1.864 (1.453,2.392)**
Gout	1068 (1.2)	18614 (0.6)	**1.950 (1.833,2.074)**	1062 (1.2)	859 (1.0)	**1.273 (1.164,1.393)**
Connective tissue diseases						
Systemic lupus erythematosus	165 (0.2)	2837 (0.1)	**1.972 (1.685,2.307)**	161 (0.2)	101 (0.1)	**1.639 (1.278,2.102)**
Sjögren syndrome	232 (0.3)	2906 (0.1)	**2.706 (2.367,3.093)**	226 (0.3)	147 (0.2)	**1.582 (1.285,1.947)**
Dermatopolymositis	23 (<0.1)	366 (<0.1)	**2.133 (1.400,3.250)**	21 (<0.1)	17 (<0.1)	1.271 (0.670,2.408)
Systemic sclerosis	34 (<0.1)	521 (<0.1)	**2.211 (1.563,3.129)**	34 (<0.1)	26 (<0.1)	1.344 (0.807,2.240)

TF, TF; 95% CI, 95% confidence interval; HR, hazard ratio. In the TriNetX analytics platform, deidentification rules require that any incident case count of 10 or fewer be reported as 10. In the crude model, no propensity score matching was performed. In contrast, Matching Model 1 incorporated matching based on age at the index date and sex. The bold values indicates statistically significance.

**Table 5:** Sensitivity analysis assessing the risk of incident systemic inflammatory and autoimmune rheumatic diseases in patients with trigger finger under varying trigger finger definition.

Outcomes	Definition 1		
	TF cohort (%)	Control cohort (%)	HR (95% CI)
Inflammatory arthritis			
Ankylosing spondylitis	NA		
Rheumatoid arthritis	30 (0.1)	30 (0.1)	**2.524 (1.649,3.864)**
Psoriatic arthritis	25 (0.1)	25 (0.1)	**2.524 (1.583,4.024)**
Gout	287 (1.2)	287 (1.2)	**1.234 (1.054,1.444)**
Connective tissue diseases			
Systemic lupus erythematosus	46 (0.2)	46 (0.2)	1.212 (0.816,1.799)
Sjögren syndrome	48 (0.2)	48 (0.2)	**1.577 (1.095,2.272)**
Dermatopolymositis	NA		
Systemic sclerosis	NA		

TF, trigger finger; 95% CI, 95% confidence interval; HR, hazard ratio. In the TriNetX analytics platform, deidentification rules require that any incident case count of 10 or fewer be reported as 10.

In this model, patients were included in the TF group only when both conditions were met: a recorded diagnosis of trigger finger (ICD-10-CM M65.3) and record of injection of tendon sheath/ligament (CPT code: 20550). A 1:1 propensity score–matched design was implemented in each analysis. Variables accounted for in the adjustment comprised demographics (age, sex, race, body mass index), healthcare use setting (outpatient and inpatient encounters), coexisting comorbidities (hypertension, hyperlipidemia, diabetes mellitus, chronic ischemic heart disease, vitamin D deficiency, chronic kidney disease, liver disorders), concurrent comedications (glucocorticoids, nonsteroidal anti-inflammatory agents), socioeconomic factors (individuals facing health risks related to socioeconomic and psychosocial conditions), substance-related status (mental and behavioral disorders associated with psychoactive substance use), and familial history of arthritis and other disorders of the musculoskeletal system and connective tissue. The bold values indicates statistically significance.

### Stratification analyses

In age-stratified analyses, the association between TF and systemic rheumatic diseases was observed in both age groups, with generally stronger effect sizes among individuals aged 18–64 years (**Table 6**). In this younger group, TF was associated with increased risks of ankylosing spondylitis (HR: 2.301; 95% CI: 1.338–3.955), rheumatoid arthritis (HR: 2.060; 95% CI: 1.456–2.915), and psoriatic arthritis (HR: 2.689; 95% CI: 1.891–3.824). Similarly, elevated risks were observed for systemic lupus erythematosus (HR: 1.998; 95% CI: 1.415–2.823) and Sjögren syndrome (HR: 1.945; 95% CI: 1.354–2.793). However, the association with gout was not statistically significant (HR: 1.139; 95% CI: 0.958–1.353). Among patients aged >65 years, TF remained associated with increased risks of ankylosing spondylitis (HR: 2.300; 95% CI: 1.340–3.946), rheumatoid arthritis (HR: 1.556; 95% CI: 1.151–2.103), and psoriatic arthritis (HR: 1.468; 95% CI: 1.030–2.090), Gout demonstrated a modest but significant increase in this older population (HR: 1.145; 95% CI: 1.028–1.274). The risk of systemic lupus erythematosus also remained elevated (HR: 1.727; 95% CI: 1.185–2.517), whereas the association with Sjögren syndrome was not statistically significant (HR: 1.216; 95% CI: 0.948–1.560).

**Table 6:** Age-stratified risk of incident systemic inflammatory and autoimmune rheumatic diseases among patients with TF.

Outcomes	18–64 years old			Greater than 65 years old		
	TF cohort (%)	Control cohort (%)	HR (95% CI)	TF cohort (%)	Control cohort (%)	HR (95% CI)
Inflammatory arthritis						
Ankylosing spondylitis	42 (0.1)	19 (0.1)	**2.301 (1.338,3.955)**	43 (0.1)	19 (<0.1)	**2.300 (1.340,3.946)**
Rheumatoid arthritis	95 (0.3)	48 (0.1)	**2.060 (1.456,2.915)**	107 (0.2)	70 (0.1)	**1.556 (1.151,2.103)**
Psoriatic arthritis	111 (0.3)	43 (0.1)	**2.689 (1.891,3.824)**	75 (0.2)	52 (0.1)	**1.468 (1.030,2.090)**
Gout	270 (0.7)	247 (0.7)	1.139 (0.958,1.353)	712 (1.4)	634 (1.3)	**1.145 (1.028,1.274)**
Connective tissue diseases						
Systemic lupus erythematosus	94 (0.3)	49 (0.1)	**1.998 (1.415,2.823)**	73 (0.1)	43 (0.1)	**1.727 (1.185,2.517)**
Sjögren syndrome	84 (0.2)	45 (0.1)	**1.945 (1.354,2.793)**	136 (0.3)	114 (0.2)	1.216 (0.948,1.560)
Dermatopolymositis	NA			12 (<0.1)	12 (<0.1)	1.111 (0.490,2.518)
Systemic sclerosis	NA			22 (<0.1)	22 (<0.1)	1.723 (0.868,3.421)

TF, TF; 95% CI, 95% confidence interval; HR, hazard ratio. In the TriNetX analytics platform, deidentification rules require that any incident case count of 10 or fewer be reported as 10.

A 1:1 propensity score–matched design was implemented in each analysis. Variables accounted for in the adjustment comprised demographics (age, sex, race, body mass index), healthcare use setting (outpatient and inpatient encounters), coexisting comorbidities (hypertension, hyperlipidemia, diabetes mellitus, chronic ischemic heart disease, vitamin D deficiency, chronic kidney disease, liver disorders), concurrent comedications (glucocorticoids, nonsteroidal anti-inflammatory agents), socioeconomic factors (individuals facing health risks related to socioeconomic and psychosocial conditions), substance-related status (mental and behavioral disorders associated with psychoactive substance use), and familial history of arthritis and other disorders of the musculoskeletal system and connective tissue. The bold values indicates statistically significance.

Sex-stratified analyses demonstrated consistent associations across both males and females, with some variation in magnitude (**Table 7**). Among males, TF was associated with an increased risk of psoriatic arthritis (HR: 2.464; 95% CI: 1.620–3.747) and gout (HR: 1.188; 95% CI: 1.056–1.337), while the association with rheumatoid arthritis did not reach statistical significance (HR: 1.473; 95% CI: 0.909–2.387). The association with Sjögren syndrome was also not significant in males (HR: 1.276; 95% CI: 0.718–2.268). In females, TF was associated with increased risks across a broader range of outcomes, including ankylosing spondylitis (HR: 2.560; 95% CI: 1.524–4.301), rheumatoid arthritis (HR: 1.730; 95% CI: 1.350–2.219), and psoriatic arthritis (HR: 1.865; 95% CI: 1.342–2.592). Elevated risks were also observed for systemic lupus erythematosus (HR: 1.537; 95% CI: 1.187–1.990) and Sjögren syndrome (HR: 1.604; 95% CI: 1.285–2.002), as well as systemic sclerosis (HR: 1.782; 95% CI: 1.013–3.133). Gout showed a modest but significant increase (HR: 1.171; 95% CI: 1.025–1.337).

**Table 7:** Sex-stratified risk of incident systemic inflammatory and autoimmune rheumatic diseases among patients with TF.

Outcomes	Male			Female		
	TF cohort (%)	Control cohort (%)	HR (95% CI)	TF cohort (%)	Control cohort (%)	HR (95% CI)
Inflammatory arthritis						
Ankylosing spondylitis	NA			50 (0.1)	20 (<0.1)	**2.560 (1.524,4.301)**
Rheumatoid arthritis	40 (0.1)	167 (0.3)	1.473 (0.909,2.387)	167 (0.3)	99 (0.2)	**1.730 (1.350,2.219)**
Psoriatic arthritis	74 (0.2)	100 (0.2)	**2.464 (1.620,3.747)**	100 (0.2)	55 (0.1)	**1.865 (1.342,2.592)**
Gout	596 (1.9)	466 (0.9)	**1.188 (1.056,1.337)**	466 (0.9)	408 (0.7)	**1.171 (1.025,1.337)**
Connective tissue diseases						
Systemic lupus erythematosus	NA			144 (0.3)	96 (0.2)	**1.537 (1.187,1.990)**
Sjögren syndrome	26 (0.1)	200 (0.4)	1.276 (0.718,2.268)	200 (0.4)	128 (0.2)	**1.604 (1.285,2.002)**
Dermatopolymositis	NA			16 (<0.1)	11 (<0.1)	1.491 (0.692,3.213)
Systemic sclerosis	NA			33 (<0.1)	19 (<0.1)	**1.782 (1.013,3.133)**

TF, TF; 95% CI, 95% confidence interval; HR, hazard ratio. In the TriNetX analytics platform, deidentification rules require that any incident case count of 10 or fewer be reported as 10.

A 1:1 propensity score–matched design was implemented in each analysis. Variables accounted for in the adjustment comprised demographics (age, sex, race, body mass index), healthcare use setting (outpatient and inpatient encounters), coexisting comorbidities (hypertension, hyperlipidemia, diabetes mellitus, chronic ischemic heart disease, vitamin D deficiency, chronic kidney disease, liver disorders), concurrent comedications (glucocorticoids, nonsteroidal anti-inflammatory agents), socioeconomic factors (individuals facing health risks related to socioeconomic and psychosocial conditions), substance-related status (mental and behavioral disorders associated with psychoactive substance use), and familial history of arthritis and other disorders of the musculoskeletal system and connective tissue. The bold values indicates statistically significance.

## Discussion

In this large-scale retrospective cohort study, TF was associated with an increased incidence of several systemic inflammatory and autoimmune rheumatic diseases. However, given the observational nature of the study, these findings should not be interpreted as evidence of a causal relationship. The temporal and biological interplay between TF and systemic rheumatic diseases remains complex. These associations were consistently identified across both the global and US cohorts, particularly for inflammatory arthritis, including ankylosing spondylitis, rheumatoid arthritis, and psoriatic arthritis, as well as selected connective tissue diseases such as systemic lupus erythematosus and Sjögren syndrome. The overall pattern of findings remained similar across multiple sensitivity analyses and stratified subgroups, although variations in effect size were noted by age and sex.

Previous studies on TF have primarily focused on its local pathology and its association with metabolic conditions, particularly diabetes mellitus, as well as its co-occurrence with established inflammatory diseases such as rheumatoid arthritis (6, 11, 12). However, evidence examining the relationship between TF and other systemic autoimmune or inflammatory rheumatic diseases remains limited. Most prior investigations have focused on single conditions, often without appropriate comparator groups or longitudinal follow-up (13). As a result, the broader relationship between TF and a spectrum of systemic rheumatic diseases has not been well characterized. In this context, the present study extends existing knowledge by systematically evaluating multiple autoimmune and inflammatory rheumatic outcomes in a large real-world cohort, while applying propensity score matching and multiple sensitivity analyses.

Several potential mechanisms may underlie the observed associations between TF and systemic autoimmune and inflammatory rheumatic diseases. We suppose chronic low-grade inflammation, immune dysregulation, and synovial involvement could contribute to tenosynovial changes, thereby increasing the likelihood of developing TF as an early or concomitant manifestation. Another possible explanation involves the interaction between mechanical stress and immune activation. Repetitive mechanical loading of the flexor pulley induces local tissue injury through biomechanical stress, particularly during high-force gripping positions such as the crimp grip in rock climbing (14, 15). This concept is could complement with the Koebner phenomenon described in psoriatic disease, in which mechanical trauma may precipitate inflammation at predisposed sites (16). Likewise, ankylosing spondylitis and psoriatic arthritis are increasingly regarded as disorders involving the synovio-entheseal complex, where inflammation may originate at the enthesis before extending to adjacent synovial structures (17). Although direct evidence linking TF to these mechanisms remains limited, the relatively stronger associations observed for ankylosing spondylitis and psoriatic arthritis in our study may be compatible with this pathogenic framework.

From a local tissue perspective, TF is characterized by thickening of the A1 pulley, fibrocartilaginous metaplasia, and increased deposition of extracellular matrix components (1, 2, 18). Although research on tendinopathy more generally points to the involvement of pro-inflammatory cytokines and various growth factors, histopathological analyses of TF specimens tend to highlight degenerative alterations, with comparatively limited evidence of a dominant inflammatory process (2, 19). Although current evidence remains insufficient to substantiate this hypothesis, we speculate that TF could reflect a localized manifestation of as-yet uncharacterized dysregulated inflammatory signaling, rather than being explained solely by mechanical factors. Metabolic influences may also further help explain this association. TF occurs more frequently in individuals with diabetes (20), in whom chronic hyperglycemia drives the accumulation of advanced glycation end products (AGEs), increased oxidative stress, and microvascular impairment (21, 22). These pathophysiological changes could potentially alter tissue properties and foster a pro-inflammatory milieu. For example, the accumulation of AGEs and heightened oxidative stress may facilitate the development of autoimmune responses by generating neoantigens, activating immune pathways through the receptor for advanced glycation end products (RAGE), and undermining mechanisms of immune tolerance. At the same time, the interplay between metabolic disturbance and autoimmunity is not unidirectional, but instead reflects a complex and reciprocal relationship (23–25). Although metabolic syndrome–related variables were included as covariates in our analysis, we postulate that underlying metabolic dysregulation may still function as a shared upstream driver linking TF with systemic inflammatory diseases. However, it is important to note that though these mechanisms are well-established in diabetic tendinopathy broadly, their specific contribution to TF pathogenesis remains to be fully elucidated in flexor tendon tissue.

Notably, gout differs from other systemic rheumatic diseases in its underlying pathophysiology. Rather than being primarily driven by autoimmune or systemic inflammatory mechanisms, gout is characterized by monosodium urate crystal deposition, which can infiltrate peri-tendinous and tenosynovial structures (26). This localized crystal deposition may lead to tendon thickening, and mechanical obstruction within the flexor tendon sheath, thereby contributing to the development of TF (27, 28). Prior surgical and pathological observations have demonstrated that tophaceous infiltration within tendon sheaths can result in triggering phenomena and impaired tendon gliding (29). Therefore, the observed association between gout and TF in this study may, at least in part, reflect a direct structural and mechanical effect rather than a shared systemic inflammatory pathway.

Although several associations reached statistical significance, the absolute event rates remained low for many outcomes. Therefore, these findings should be interpreted in the context of both relative and absolute risk. Rather than supporting routine rheumatologic screening for all patients with TF, our results suggest that clinicians should maintain awareness of the potential coexistence of systemic inflammatory and autoimmune rheumatic diseases, particularly when patients present with persistent inflammatory symptoms, multiple musculoskeletal manifestations, or other features suggestive of systemic disease.

This study has several strengths. First, we utilized a large, multinational real-world database, allowing for the inclusion of a substantial number of patients and enabling the evaluation of relatively rare outcomes. Second, the use of PSM improved comparability between cohorts by balancing a wide range of demographic and clinical variables. However, several limitations should be acknowledged. First, the use of administrative coding may introduce misclassification bias for both exposure and outcomes, and detailed clinical information such as disease severity, laboratory parameters, imaging findings, and specialist-confirmed diagnoses, were not available. Second, residual confounding from unmeasured factors remains possible. In particular, the database does not capture occupational information, such as repetitive hand use, manual labor, or workplace mechanical loading, which are recognized risk factors for TF. Likewise, detailed lifestyle behaviors, healthcare-seeking patterns, and disease detection intensity were unavailable and therefore could not be fully accounted for. Third, the temporal relationship between TF and systemic rheumatic diseases should be interpreted with caution, as reverse causation or shared underlying predisposition may contribute to the observed associations, and due to the observational cohort nature, causation could not be confirmed. Fourth, although hazard ratios were estimated using the built-in analytics function in the TriNetX platform, the proportional hazards assumption could not be formally evaluated because individual-level survival data and diagnostic testing functions are not accessible. Likewise, detailed patient-level censoring information, including loss to follow-up or transfer outside participating healthcare organizations, is unavailable within the platform. Fifth, although similar findings were observed in the TriNetX US Collaborative Network, this network is not completely independent of the Global Collaborative Network because participating US healthcare organizations contribute to both databases. Therefore, the validation analysis should be interpreted as confirmation of reproducibility within a partially overlapping network rather than a fully independent external validation. Sixth, patients with a history of malignancy were excluded from the present analysis; therefore, our findings may not be generalizable to oncology populations. Cancer patients, particularly those receiving immune checkpoint inhibitors, may exhibit a distinct spectrum of immune-related adverse events involving rheumatic, musculoskeletal, and cutaneous tissues. Autoimmune bullous dermatoses represent one well-recognized manifestation of treatment-induced immune dysregulation in this setting (30). Although there is currently no evidence directly linking immune checkpoint inhibitor therapy to TF, future studies should investigate whether the association between TF and systemic inflammatory and autoimmune rheumatic diseases differs among patients with malignancy or according to immunotherapy exposure.

In conclusion, this study identified consistent associations between TF and diagnoses of several systemic inflammatory and autoimmune rheumatic diseases in large real-world cohorts. While these findings do not establish causality, they suggest that TF may coexist with or precede a broader spectrum of inflammatory conditions. Clinically, these findings highlight the importance of considering possible underlying systemic comorbidities when evaluating patients with TF, especially when other risk factors or relevant clinical signs are present. To better delineate these associations, future research is warranted to integrate more granular clinical information alongside mechanistic studies aimed at uncovering the underlying pathways.

## Data Availability

The data analyzed in this study is subject to the following licenses/restrictions: Data in this study were retrieved from TriNetX Research Network. All data available in the database were administrated by the TriNetX platform. Detailed information can be retrieved at the official website of the research network (https://trinetx.com). Requests to access these datasets should be directed to TriNetX (https://trinetx.com).
