# Supplementary material for: Trigger finger and the risk of systemic inflammatory and autoimmune rheumatic diseases: real-world evidence
Source: Front Immunol. 2026 Aug 26;17:1872187. doi: 10.3389/fimmu.2026.1872187 (PMC13553544; doi:10.3389/fimmu.2026.1872187)
Supplement: Supplementary file 1 [file Table1.docx]

**Supplementary Tables**

**Table S1. Utilized proxy codes**

| **Description** | **ICD-10-CM code** | **VA code** |
| --- | --- | --- |
| *Study population* |  |  |
| Trigger finger | M65.3 |  |
| *Outcome: Systemic Inflammatory and Autoimmune Rheumatic Diseases* | | |
| Inflammatory arthritis | | |
| Ankylosing spondylitis | M45 |  |
| Rheumatoid arthritis with rheumatoid factor | M05 |  |
| Rheumatoid arthritis without rheumatoid factor | M06.0 |  |
| Psoriatic arthritis | L40.5 |  |
| Gout | M10 |  |
| Connective tissue disease | | |
| Systemic lupus erythematosus | M32 |  |
| Sjögren syndrome | M35.0 |  |
| Dermatopolymyositis | M33 |  |
| Systemic sclerosis | M34 |  |
| *Other diseases or covariates* | | |
| Encounter for general examination | Z00.00 |  |
| Essential hypertension | I10 |  |
| Hyperlipidemia | E78.5 |  |
| Mental and behavioral disorders due to psychoactive substance use | F10-F19 |  |
| Diabetes mellitus | E08-E13 |  |
| Vitamin D deficiency | E55 |  |
| Chronic ischemic heart disease | I25 |  |
| Chronic kidney disease | N18 |  |
| Diseases of liver | K70-K77 |  |
| Persons with potential health hazards related to socioeconomic and psychosocial circumstances | Z55-Z65 |  |
| Family history of arthritis and other diseases of the musculoskeletal system and connective tissue | Z82.6 |  |
| *Co-medications* | | |
| Glucocorticoids |  | HS051 |
| Nonsteroidal anti-inflammatory drugs |  | CN104 |

**Table S2. Description of all applied sensitivity analysis models**

| **Models** | **Description** | **Corresponding tables** |
| --- | --- | --- |
| **Applying different follow-up time after index date** | | |
| 10 years /15 years after index date | The analysis considers only those outcome events occurring within the specified follow-up window. In addition, all sub-analyses applied a uniform three-month washout interval. | **Table 2** |
| **Applying different wash-out period after index date** | | |
| 12 months/24 months after index date | Events that arise within the defined washout interval are omitted from the analysis. Across all sub-analyses, participants were then observed for outcomes over a 15-year follow-up period. | **Table 3** |
| **Applying different matching covariates** | | |
| Crude model | No propensity score matching was applied in this analysis. | **Table 4** |
| Matching model 1 | The variables used for matching were age at the index date and sex. |  |
| **Applying different definition of TF** | | |
| Definition 1 | Patients were included in the TF group only when both conditions were met: a recorded diagnosis of trigger finger (ICD-10-CM M65.3) and record of injection of tendon sheath/ligament (CPT code: 20550). | **Table 5** |

**Table S3.** Negative outcome analysis in TF patients comparing with TF-free controls

| Negative outcomes | Hazard Ratio (95% CI)^a^ |
| --- | --- |
| Infectious mononucleosis | 0.708 (0.433,1.156) |
| Acute appendicitis | 0.982 (0.786,1.227) |

^a^ A 1:1 propensity score–matched design was implemented.

Variables accounted for in the adjustment comprised demographics (age, sex, race, body mass index), healthcare use setting (outpatient and inpatient encounters), coexisting comorbidities (hypertension, hyperlipidemia, diabetes mellitus, chronic ischemic heart disease, vitamin D deficiency, chronic kidney disease, liver disorders), concurrent comedications (glucocorticoids, nonsteroidal anti-inflammatory agents), socioeconomic factors (individuals facing health risks related to socioeconomic and psychosocial conditions), substance-related status (mental and behavioral disorders associated with psychoactive substance use), and familial history of arthritis and other disorders of the musculoskeletal system and connective tissue.

**Table S4.** Evaluating the risk of seronegative rheumatoid arthritis in TF patients comparing with TF-free controls

| Negative outcomes | Hazard Ratio (95% CI)^a^ |
| --- | --- |
| Rheumatoid arthritis without rheumatoid factor | 2.945 (2.244,3.864) |

^a^ A 1:1 propensity score–matched design was implemented; Rheumatoid arthritis without rheumatoid factor was set as ICD-10-CM code M06.0

Variables accounted for in the adjustment comprised demographics (age, sex, race, body mass index), healthcare use setting (outpatient and inpatient encounters), coexisting comorbidities (hypertension, hyperlipidemia, diabetes mellitus, chronic ischemic heart disease, vitamin D deficiency, chronic kidney disease, liver disorders), concurrent comedications (glucocorticoids, nonsteroidal anti-inflammatory agents), socioeconomic factors (individuals facing health risks related to socioeconomic and psychosocial conditions), substance-related status (mental and behavioral disorders associated with psychoactive substance use), and familial history of arthritis and other disorders of the musculoskeletal system and connective tissue.
